# Effectiveness of Mobile Apps in Improving Medication Adherence Among Chronic Kidney Disease Patients: Systematic Review

**DOI:** 10.2196/53144

**Published:** 2025-04-16

**Authors:** Ganesh Sritheran Paneerselvam, Pei Lin Lua, Wen Han Chooi, Inayat Ur Rehman, Khang Wen Goh, Long Chiau Ming

**Affiliations:** 1 School of Pharmacy Taylor's University Subang Jaya Malaysia; 2 Digital Health and Innovation Impact Lab Taylor's University Subang Jaya Malaysia; 3 Faculty of Pharmacy Universiti Sultan Zainal Abidin Besut Malaysia; 4 Faculty of Medicine Quest International University Perak Malaysia; 5 Department of Clinical Pharmacy and Pharmacy Practice Faculty of Pharmacy Universiti Malaya Kuala Lumpur Malaysia; 6 Faculty of Data Science and Information Technology INTI International University Nilai Malaysia; 7 Sir Jeffrey Cheah Sunway Medical School Faculty of Medical and Life Sciences Sunway University Sunway City Malaysia; 8 Datta Meghe College of Pharmacy Datta Meghe Institute of Higher Education and Research (deemed to be University) Wardha India

**Keywords:** mobile applications, medication adherence, chronic kidney disease, health outcomes, mobile health, mhealth, digital health, kidney disease, patient education, medication monitoring, e-medication, electronic medication

## Abstract

**Background:**

Chronic kidney disease (CKD) is a serious condition affecting millions of individuals worldwide. Adherence to medication regimens among patients with CKD is often suboptimal, leading to poor health outcomes. In recent years, mobile apps have gained popularity as a promising tool to improve medication adherence and self-management in various chronic diseases.

**Objective:**

This study aimed to evaluate the effectiveness of mobile apps to improve medication adherence among patients with CKD (including end-stage and renal replacement therapy).

**Methods:**

A systematic search was conducted using Scopus, Cochrane, PubMed, and EBSCOhost to include eligible articles that studied mobile apps to improve medication adherence among patients with CKD. The quality of the selected studies was evaluated using the Newcastle‒Ottawa Scale and the Cochrane risk-of-bias tool.

**Results:**

Out of 231 relevant articles, only 9 studies were selected for this systematic review. Based on Newcastle‒Ottawa Scale, 7 were deemed to be of high quality, while others were of fair quality. The Cochrane risk-of-bias tool indicated a low to moderate risk of bias across the included studies. Most of the included studies had a randomized controlled design. Of the 9 selected studies, 3 papers represented medication adherence by a coefficient of 10 variability of tacrolimus, 3 papers used adherence measurement scales to calculate the score for assessing medication adherence, 2 papers represented medication adherence by self-reporting, 2 papers represented medication adherence using electronic monitoring, and 1 represented medication adherence by pill count. The mobile apps were identified as Transplant Hero (Transplant Hero LLC), Perx (Perx Health), Smartphone Medication Adherence Saves Kidneys (developed by John McGillicuddy), Adhere4U (developed by Ahram Han), My Dialysis (developed by Benyamin Saadatifar), Kidney Love (developed by National Kidney foundation), and iCKD (developed by Dr Vivek Kumar). Of these apps, 3 focused on evaluating Transplant Hero, while the remaining investigated each of the other mentioned apps individually. The apps use various strategies to promote medication adherence, including reminders, gamification, patient education, and medication monitoring. A majority, 5 out of 9 mobile apps, had a statistically significant (*P*<.05) effect on medication adherence. There was strong evidence for a positive effect of interventions focusing on games and reminders combined with electronic medication tray monitoring and patient education.

**Conclusions:**

Mobile apps effectively improved medication adherence in patients with CKD, but low evidence and short intervention duration warrant caution. Future research should identify ideal features, provider costs, and user-friendly, secure apps.

## Introduction

Chronic kidney disease (CKD) is a global health challenge affecting millions and contributing to millions of premature deaths worldwide [1]. Managing CKD involves long-term care, with medication adherence being a pivotal aspect [2]. Unfortunately, patients with CKD often struggle with medication adherence, leading to adverse health outcomes and increased health care costs [3-5]. Several factors contribute to nonadherence, such as complex medication regimens, forgetfulness, lack of understanding about the importance of adherence, and limited patient-provider communication [6]. Nonadherence to medication in CKD can further exacerbate the progression of the disease, resulting in complications and compromised quality of life for patients [7,8].

In recent years, mobile apps have gained popularity in health care, offering a promising solution to enhance medication adherence and empower patients to manage their chronic conditions [9,10]. These apps provide a comprehensive toolkit, including medication reminders, tracking mechanisms, educational resources, and communication platforms, designed to improve patient adherence [11-13]. CKD is an umbrella term that encompasses a range of conditions affecting the kidneys, including end-stage conditions that necessitate treatments such as dialysis and renal transplantation [2].

Despite the growing body of research on the efficacy of mobile apps in fostering medication adherence for various chronic diseases, there is a noticeable gap in the literature regarding their application in CKD management [14]. The unique challenges and complexities of CKD warrant a specialized investigation into the role of mobile apps in enhancing medication adherence among this specific patient group [13]. This systematic review aims to fill this knowledge gap by rigorously assessing the effectiveness of mobile apps in improving medication adherence among patients with CKD [6,15,16]. By systematically evaluating the impact of mobile apps on medication adherence in CKD, this review not only illuminates the current state of the field but also provides insights that can guide the development of future interventions [15-17]. These findings have the potential to optimize patient care and alleviate the substantial health care burden associated with CKD. This study aims to evaluate the effectiveness of mobile apps to improve medication adherence among patients with CKD.

## Methods

### Search Strategy

This is a systematic review that analyzed articles that fulfill the specified criteria to evaluate the effectiveness of mobile apps to improve medication adherence in the CKD population (which includes patients with hemodialysis, peritoneal dialysis, and renal transplant). Between January and April 2023, a literature search was conducted using 4 search engines, namely, Scopus, Cochrane CENTRAL, PubMed, and EBSCOHost. The following search terms were used to find article titles using Boolean operators: (digital OR mobile OR application OR app OR smartphone) AND (“kidney disease” OR “renal disease” OR “CKD” or “dialysis” OR “renal replacement therapy” OR “haemodialysis” OR “hemodialysis” OR “kidney transplant” OR “renal transplant” OR “kidney failure” OR “renal failure” OR “ESRD” OR “ESKD”) AND (“medication adherence”).

### Inclusion and Exclusion Criteria

The inclusion criteria focused on quantitative studies, such as randomized controlled trials (RCTs), cohort studies, cross-sectional studies, and case-control studies, involving patients with CKD of all age groups, including those undergoing dialysis and kidney transplant recipients. Only studies written in English and accessible in full text were considered to ensure clarity and comprehensibility. The primary intervention of interest was mobile apps specifically designed for managing medication adherence in patients with CKD, though apps with additional functionalities were also included if they addressed this focus. While, all systematic literature reviews, pilot studies, and abstracts were excluded. Non-English publications and studies that did not clearly specify the use of mobile apps for CKD medication adherence were also excluded.

### Study Population

This systematic review focused on adult patients aged 18 years and older who are diagnosed with CKD. The study population included individuals undergoing various stages of CKD management, specifically those receiving hemodialysis, peritoneal dialysis, or those who had undergone a kidney transplant. To ensure a relevant and specific analysis, the review exclusively included studies that investigated mobile apps aimed at enhancing medication adherence within these distinct patient groups.

### Study Selection

The study selection process involved several systematic steps to ensure a comprehensive and unbiased review of the literature. The process began by identifying potentially relevant articles using their titles and abstracts based on the search strategy’s key terms, duplicates were detected and eliminated using EndNote (Clarivate) reference management software, which automatically identified duplicates based on title, author, and publication year. Manual verification was performed to ensure accuracy. The screening process was conducted in 2 stages. In the first stage, the eligibility of the articles was assessed by evaluating the titles and abstracts independently by 2 authors (IUR and KWG). The full text of each of the retrieved articles was independently screened using predefined inclusion and exclusion criteria. Articles that did not meet the eligibility criteria were excluded from the study. In cases where multiple publications of the same study were found, only publications containing fully published results were included. To ensure the reliability of the screening process, at least 2 independent raters performed the review. Discrepancies between the raters were resolved through discussion and consensus. In cases where consensus could not be reached, a third author was consulted to provide an additional perspective and assist in resolving the discrepancy. This method ensured the robustness and objectivity of the study selection process. In total, 63 articles were excluded during the abstract screening phase, as they either did not align with the study objectives or lacked the necessary data on medication adherence in patients with CKD through mobile apps. While 19 articles were excluded due to irrelevance to the primary focus of the review, insufficient detail on mobile app usage specific to patients with CKD, or failure to meet the predefined inclusion criteria (Multimedia Appendix 1 for the PRISMA [Preferred Reporting Items for Systematic Reviews and Meta-Analyses] checklist).

### Quality Assessment

The quality of the selected studies was evaluated using the Newcastle‒Ottawa Scale, and the Cochrane risk-of-bias tool. It is a tool recommended for evaluating nonrandomized studies such as case-control and cohort studies, as well as customized for cross-sectional studies. Discrepancies in the quality ratings were resolved through discussion and consensus between the 2 authors (IUR and KWG). In instances where consensus could not be achieved, a third author was consulted to provide an additional perspective and aid in resolving the discrepancy. This approach ensured a robust and reliable assessment of the quality of the included studies. This scale uses a “star scoring system” with a maximum of 9 points to measure bias across 3 domains: participant selection and exposure measurement (up to 4 points), comparability of groups (up to 2 points), and result in adequacy, follow-up, or ascertainment of exposure or outcome (up to 3 points). Studies with ≥7 stars were considered high quality, 2 to 6 points indicated fair quality, and ≤1 point signified poor quality [18,19].

### Risk of Bias Assessment

We used the Cochrane risk-of-bias tool to assess the quality of the 3 RCTs included in our systematic review. This tool evaluates the domains mentioned in Textbox 1.

Each study was independently assessed by 2 authors (IUR and KWG), and any discrepancies were resolved through discussion and consensus.

**Domains were assessed using the Cochrane risk-of-bias tool.**

- Random sequence generation
- Allocation concealment
- Blinding of participants and personnel
- Blinding of outcome assessment
- Incomplete outcome data
- Selective reporting
- Other sources of bias

### Data Synthesis

Data synthesis involved a comprehensive approach to aggregate and interpret the findings from the included studies. Initially, we extracted key data from each study, including study design, sample size, duration, participant characteristics, type of mobile app used, methods to measure adherence and outcomes. Quantitative data were synthesized using descriptive statistics, and results were presented in tables to facilitate comparison across studies. For studies reporting on medication adherence outcomes, we calculated effect sizes where possible and examined the statistical significance of the reported results. We also performed a narrative synthesis to qualitatively summarize the findings, highlighting common themes, and variations in the effectiveness of the mobile apps on medication adherence among patients with CKD. Where appropriate, we categorized the studies based on the type of mobile app interventions and analyzed the outcomes within these categories to identify trends and draw conclusions about the efficacy of different app features.

## Results

### General Study Characteristics

**Figure 1:**
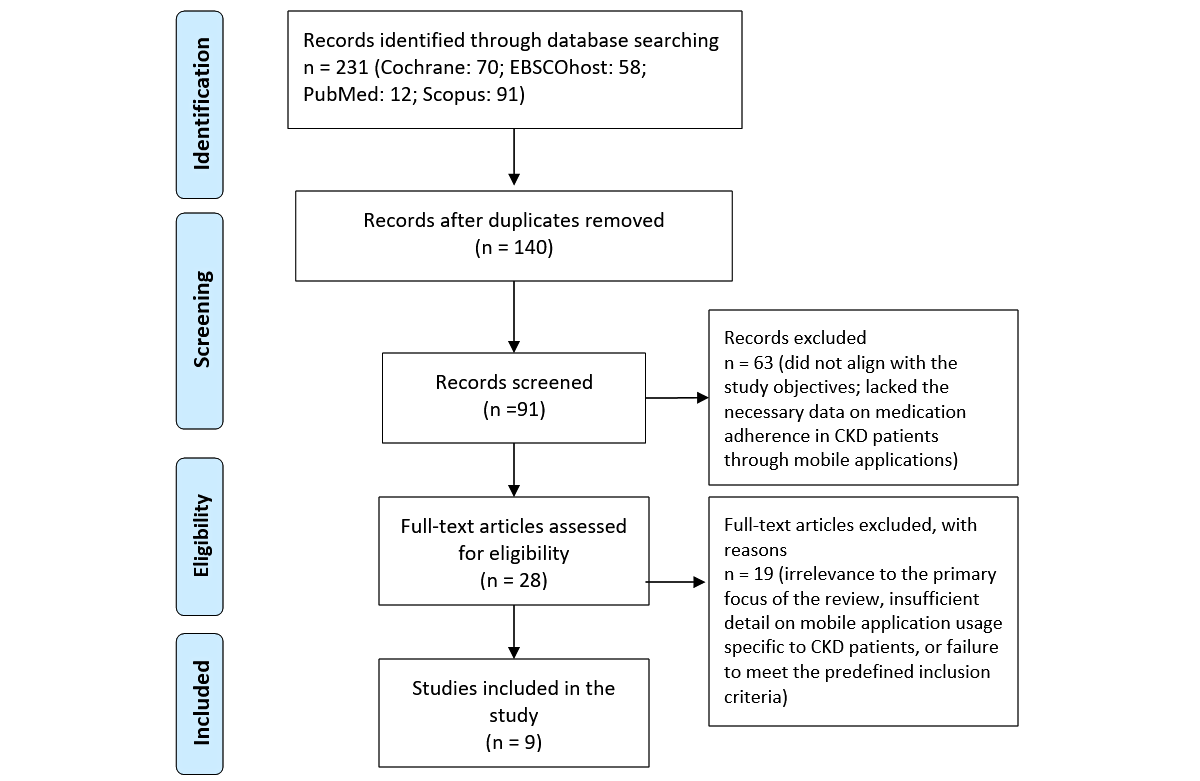
Summary of the article selection process.

All 9 included studies were published between 2017 and 2023. A summary of the article selection process is shown in Figure 1 All included papers were also conducted in various countries, which provides the benefits of gathering relevant information from different countries and cultures. Among 9 of the studies, 4 were conducted in the United States, 2 were conducted in South Korea, and 1 study each was performed in Australia, Iran, and Taiwan. Most of the included studies (n=3) were a randomized controlled design, 2 were conducted in a prospective cohort design, 2 were in a quasi-experimental study and 2 were carried out in an observational study design. In addition, the sample size varied in all studies from a minimum of 67 participants to a maximum of 214 participants. Thus, a total of 1023 participants were included in this study. Furthermore, most of the studies had both male and female participants. However, some of them did not consider gender when choosing their participants’ characteristics. Next, the age group selected in all studies varied, but the mean age concluded from all papers was 52.65 (SD 5.93) years old. Patients diagnosed with CKD, undergoing hemodialysis or peritoneal dialysis, or who received a kidney transplant were selected by all the studies. Among all 9 papers, 5 selected research articles selected patients with renal transplants, 2 studies specifically selected patients with CKD without specifying whether they were undergoing renal replacement therapy, and 2 studies included patients with hemodialysis. The majority of the patients who participated were male, with a percentage of 65.17% (270/414). The characteristics of the included studies are represented in Table 1.

**Table 1:** Demographic characteristics of the selected studies.

	Author, year and country	Study design and sample size	Study duration	Participant characteristics
1.	Levine et al [20], United States	Prospective cohort study (N=108)	3 months	Renal transplant recipients.
2.	Li et al [21], Australia	Randomized controlled trial (N=6)	12 months	The participants included were between the ages of 18 and 75 diagnosed with chronic kidney disease.
3.	McGillicuddy et al [22], United States	Randomized controlled clinical trial (N=80)	12 months	Kidney transplant recipients who were identified as having both poorly controlled blood pressure and poor medication adherence.
4.	Han et al [23], South Korea	Randomized controlled study (N=136)	6 months	Renal transplant recipients taking tacrolimus or cyclosporine twice daily.
5.	Torabi et al [24], United States	Observational study (N=67)	3 months	Renal transplant recipients.
6.	Zanetti-Yabur et al [25], United States	Observational study (N=74)	3 months	Renal transplant recipients.
7.	Saadatifar et al [26], Iran	Quasi-experimental study (N= 136)	3 months	All patients undergoing hemodialysis.
8.	Park and Kim [17], South Korea	Quasi experimental study (N=84)	2 months	All patients undergoing hemodialysis.
9.	Tsai et al [27], Taiwan	Prospective cohort study (N=214)	3 months	Patients with CKD stages 1-5 who were not undergoing renal replacement therapy.

### Characteristics of Mobile Apps

The mobile apps examined in the study (n=7) were identified as Transplant Hero, Perx, Smartphone Medication Adherence Saves Kidneys (SMASK), Adhere4U, My Dialysis, Kidney Love, and iCKD. The apps used various strategies to promote medication adherence, including reminders, gamification, patient education, and medication monitoring. In total, 4 apps featured medication reminders through notifications, and one of them could be paired with a smartwatch to serve as an electronic alarm for reminders. In addition, 2 apps gamified the medication adherence process by providing digital awards and positive reinforcement for timely medication intake. Moreover, 5 apps incorporated educational components to enhance users’ knowledge about their medications, chronic kidney failure, and disease management. Furthermore, one app could be used in conjunction with an electronic medication tray to monitor adherence. Alongside these functionalities, the apps also offered unique features such as trend monitoring, motivational messages, and interpretation of examination results.

### Description of Each Mobile App

#### Transplant Hero

Transplant Hero, a mobile app designed to support patients with transplants in maintaining their medication adherence. The app offered features such as customizable medication lists, alarm reminders, adherence tracking, educational resources on transplant surgery, connections with fellow patients with transplants, sharing medication data with health care providers, custom reports, and goal-setting for medication adherence. It could also sync with smartwatches for scheduled reminders and incentivize medication adherence with digital awards. Transplant Hero was compatible with both iOS and Android devices, providing a comprehensive solution for patients with transplants to stay on track with their medication regimens [20,24,25].

#### Perx

Perx was a mobile app developed by Perx Health with the purpose of assisting individuals with chronic conditions in monitoring their medication adherence and earning rewards for their efforts. The app achieved this by offering several user-friendly features, including customizable medication lists, medication reminders, access to educational resources related to chronic disease management, opportunities to earn rewards for medication adherence, and prompts for health care visits [21].

#### SMASK

SMASK is a mobile app developed by the Medical University of South Carolina, designed to assist kidney transplant recipients in monitoring their medication adherence and maintaining blood pressure control. The app facilitated this through a range of features, allowing users to easily track their medications and blood pressure while receiving medication reminders. These features encompassed the integration of an electronic medication tray as an alarm, tracking of adherence over time, medication reminders, a blood pressure monitor for monitoring at home, reminders for using the blood pressure monitor, and the delivery of personal motivational messages to encourage adherence [22].

#### Adhere4U

Adhere4U aimed to achieve improved adherence through a range of user-friendly features, including various types of reminders for taking immunosuppressants (such as medication tapers, nondaily administration, and start-stop dates), a personal medication list with adherence tracking capabilities over time, educational resources related to immunosuppressants, and a feature for storing patients’ lab results. Notably, the app’s medication reminders and data storage functions were designed to function even without an internet connection [23].

#### My Dialysis

My Dialysis is a mobile app designed to aid patients with hemodialysis in monitoring treatment adherence. It uses video clips to educate users on topics such as kidney failure, fluid intake limitations, medication schedules, and physical activity [26].

#### Kidney Love

Kidney Love is a mobile app designed to assist kidney transplant recipients in improving self-management through dietary restrictions, blood pressure management, medication adherence, and exercise, using smartphone SMS and face-to-face counseling and education.

#### iCKD

iCKD provides tracking alerts, reminders, mobile health (mHealth) management, and personalized health education videos. The app also allows clinical physicians to remotely analyze patients’ health status through self-recorded data, enabling health care teams to provide timely feedback and reminders.

### Medication Adherence

Out of the 9 selected studies, 7 used objective measures of adherence, such as pill counting, coefficient of variability for immunosuppressants, electronic monitoring, and adherence scales. Two studies relied solely on subjective measures, using self-report questionnaires. One study used 2 objective measures along with self-reporting, while another study combined both subjective and objective measures. Overall, 5 out of the 9 studies demonstrated at least one statistically significant measure of medication adherence in patients with CKD due to the intervention of a mobile app. The summary of the findings of the types of mobile apps and their outcomes are summarized in Table 2.

**Table 2:** Summary of mobile apps used in the selected studies.

	Author, year and country	Mobile app name and uses	Method to measure adherence	Outcomes
1.	Levine et al [20], United States	Transplant Hero Uses: paired with a smartwatch to act as electronic alarm as reminder to take medications and provide patient education	CV^a^ of Tacrolimus	CV levels did not show an increase in medication adherence in all groups (not significant; *P*>.05) when tested at either one or 3 months.
2.	Li et al [21], Australia	Perx Uses: personalized notifications and an engaging game designed to encourage medication adherence. Customizable medication schedule information, educational messages on disease management, and reminders for health care appointments were included	Pill counts for overall medication adherence rate	There was statistically significant improvement in medication adherence among patients with kidney disease from the Perx group compared to the control group at month 6 (95% CI 0.02 to 0.24; *P*=.02) and month 12 (95% CI 0.01 to 0.20; *P*=.028).
3.	McGillicuddy et al [22], United States	SMASK^b^ Uses: an application featuring reminder functions integrated with an electronic medication tray for adherence tracking, complemented by the delivery of personalized motivational messages aimed at fostering medication adherence	Average medical regimen adherence using timestamp of opening pill tray and CV	Average medical regimen adherence was significantly higher compared to the attention control group (*P*<.001) at every assessment throughout the 6-month study. Patients assigned to intervention group experienced significant decrease in the average 12-month tacrolimus CV (*P*=.05) and a significant increase in the proportion of patients achieving a low tacrolimus CV (tacrolimus CV<40%; *P*=.001) in comparison to the control group.
4.	Han et al [23], South Korea	Adhere4U Uses: delivers medication intake reminders, tracks patterns in medication usage, and offers information on immunosuppressants for kidney transplant recipients.	Electronic monitoring of nonadherence rate using pill bottle and Self-reported rate of nonadherence	The overall nonadherence rate was 63.6% (75/118), and there was no significant difference observed between the 2 groups throughout the 6-month study period. Specifically, the nonadherence rates were 65.% (39/60) for the mobile group and 62.1% (36/58) for the control group, resulting in an absolute risk reduction of –2.9% (–3/118). The OR^c^ was 1.14 with a (95% CI 0.53 to 2.40).
5.	Torabi et al [24], United States	Transplant Hero Uses: paired with a smartwatch to act as electronic alarm as reminder to take medications and provide patient education.	CV of Tacrolimus	CV significantly decreased among app users compared to nonusers at 1 month (27.7 vs 37.0, respectively, *P*=.01) but had no change at 3 months (33.6 vs 35.4, respectively; *P*=.63).
6.	Zanetti-Yabur et al [25], United States	Transplant Hero Uses: an alarm system that reminds users to take their medication on time. The software is also a user-friendly, engaging, and educational tool that provides positive. Reinforcement to users for their adherence to medication.	Self-assessment using MMAS-8^d^ and IAT^e^	No significant difference (*P*>.05) was observed in the mean MMAS-8 score between users and nonusers at the 3-month mark (0.84 and 0.74, respectively). MMAS-8 scores indicated a moderate to high tendency for adherence. IAT at the 3-month point. Show users tended to achieve higher scores compared to nonusers indicating better memory of their immunosuppressive regimen, this finding did not reach statistical significance (2.25 and 1.73, respectively; *P*=.19).
7.	Saadatifar et al [26], Iran	My Dialysis Uses: application-based training program using video clips that concentrates on educating users about the kidney and its chronic failure, limitations on fluid intake, adherence to medication schedules, as well as the importance of exercise and physical activity.	ESRD-AQ^f^	Both the control group and the intervention group showed a significant increase in their treatment adherence scores after the intervention (*P*<.05). ANCOVA revealed a significant difference in treatment adherence scores between the 2 groups post intervention (*P*<.05). Interestingly, contrary to expectations, the control group, which received routine training from healthcare staff, had higher treatment adherence scores compared to the mobile health training group.
8.	Park and Kim [17], South Korea	Kidney Love Uses: encourages users to develop self-awareness, acquire knowledge and skills, enhance self-efficacy, and actively engage in their own care by encompassing multiple domains of self-management. These domains include dietary and water restriction, blood pressure management, arteriovenous fistulas, medication adherence, and exercise.	Compliance of patient role behavior tool	Experimental group demonstrated a substantial increase in treatment compliance (mean of 11.57, SD 7.63) compared to the control group (mean of –1.74, SD 2.71). This difference was highly significant (*P*<.05).
9.	Tsai et al [27], Taiwan	iCKD Uses: 10 key functionalities, which consist of tracking alerts, reminding users of outpatient visits, facilitating mobile health management, interpreting examination results, providing tailored health education videos, offering nutritional feedback and analysis, sending care and treatment notifications, providing audio responses, presenting a personalized dashboard, and integrating care quality analysis.	CKDSC^g^	Medication adherence showed no significant difference between the control and intervention group after 3 months of the study (*P*>.05).

^a^CV: Coefficient of Variability.

^b^SMASK: Smartphone Medication Adherence Saves Kidneys.

^c^OR: odds ratio.

^d^MMAS-8: Morisky Medication Adherence Scale.

^e^IAT: Immunosupp-ression Assessment Test.

^f^ESRD-AQ: End-Stage Renal Disease Adherence Questionnaire.

^g^CKDSC: chronic kidney disease Self-Care scale.

## Discussion

### Principal Findings

A majority, 5 out of 9 mobile apps, had a statistically significant (*P*<.05) effect on medication adherence. There was strong evidence for a positive effect of interventions focusing on games and reminders combined with electronic medication tray monitoring and patient education. Although Transplant Hero was the most frequently studied app (3/9), there is differing evidence regarding the productiveness of the app. Overall, this review shows that mobile apps can be effective in improving medication adherence for patients with CKD. Interventions focusing on games, reminders, and patient education have shown a positive effect on improving medication adherence among patients with CKD.

The integration of mobile apps with existing electronic health record (EHR) systems or health information technologies is a critical consideration for their successful adoption in clinical practice. Some apps, such as iCKD and Transplant Hero, offer functionalities that allow for sharing patient data with health care providers, which could potentially facilitate data integration into clinical workflows. However, the reviewed studies did not extensively document how these apps address challenges related to interoperability with EHR systems, data privacy, and security concerns. Ensuring that mobile apps comply with privacy regulations and developing standardized interfaces for better data integration could enhance their utility in clinical settings and improve patient care.

First, interventions that incorporate games have been found to be effective in improving medication adherence. A study by Pouls et al [28] demonstrated the efficacy of gaming interventions in improving medication adherence, especially when delivered through mobile apps. The use of serious games and digital eHealth interventions has shown promise in improving medication adherence in patients with rheumatoid arthritis [28]. However, it should be noted that the application of serious games in improving medication adherence requires further development [28].

Research has indicated the value of medication reminder applications in improving medication adherence among patients with CKD [29]. Santo et al [30] conducted a randomized clinical trial focusing on patients with coronary heart disease, revealing that medication reminder apps significantly enhanced adherence compared to standard care. Similarly, a study by Phutthinart et al [31] demonstrated the effectiveness of a drug reminder mobile phone app in promoting adherence to uric acid-lowering therapy in patients with gout. These findings suggest that mobile apps equipped with reminders can effectively support medication adherence among patients with CKD. Furthermore, patient education emerges as a pivotal element within interventions aimed at enhancing medication adherence. In a study by Ogedegbe et al [32], interventions that amalgamated behavioral strategies with patient education were identified as the most efficacious in improving medication adherence among individuals with chronic diseases. These patient education interventions not only addressed medication-related concerns but also provided emotional support and bolstered patients' confidence in surmounting adherence obstacles, ultimately leading to substantial improvements in adherence behaviors [33].

Moreover, factors associated with participants, such as the burden of disease, behaviors of noncompliance, perceived advantages of the medication, and potential side effects, might have contributed to differences in the observed effect sizes across studies. In addition, the variability in features and content of mobile apps could have led to a higher level of variation in their constructiveness. At present, there are no established regulatory guidelines for ensuring the quality of features and content in these apps. Consequently, most medical apps raise 2 potential issues: first, the reliability and safety of the information provided by the app cannot be guaranteed, and second, there are concerns about the privacy of patient data shared by these apps [2]. Further investigation is needed to examine the content of apps, and regulations should be implemented to safeguard patient privacy. In addition, our findings from this study indicate that a higher level of media literacy can enhance treatment adherence, as stated by Falsafi [34]. For instance, in this study conducted by Saadatifar et al [26], patients with a higher level of media literacy, who were proficient in using mobile phones and possessed moderate media literacy, demonstrated greater treatment adherence scores compared to similar studies.

Several studies in our review applied theoretical frameworks to assess the impact of mHealth apps on medication adherence. For example, the Health Belief Model was used to understand how perceived severity and susceptibility to health conditions influence adherence behavior. The Health Belief Model suggests that individuals are more likely to engage in health-promoting behaviors if they believe they are at risk and that the behavior will reduce their risk. In addition, Self-Determination Theory was used to evaluate how mobile apps foster intrinsic motivation for adherence through autonomy, competence, and relatedness. These theories provide valuable insights into how mobile apps can be designed to support behavior change and improve adherence by addressing motivational and psychological factors. However, it is important to acknowledge that mHealth interventions may encounter challenges associated with a “digital divide” among different populations based on factors such as age, education levels, and baseline self-efficacy [13,35]. Older patients with CKD may have less motivation and ability to use mHealth apps than younger patients with CKD. In addition, patients with CKD with higher education levels may derive greater benefits from mHealth interventions for CKD care [27]. This relationship between health literacy and the use of smartphones and the internet has been explored. Low health literacy was not significantly associated with a lower likelihood of owning smartphones or using the internet, but it was linked to a higher likelihood of requiring assistance with internet-based tasks [36,37]. This suggests that while health literacy may not hinder smartphone ownership or internet usage, it can affect the ability to navigate and effectively use digital resources.

Next, it is important to consider the individual characteristics and needs of patients with CKD when implementing mHealth interventions. Personalized approaches to these interventions, such as considering personality traits, have been shown to influence the adoption and active utilization of mHealth apps among patients with diabetes [10,33,38]. This is demonstrated by 3 studies that included features in their mobile apps to send personal motivational messages [22], have their personal medication lists combined with adherence tracking over time [23], and health care team monitoring via the patient’s self-recorded data on the app’s personalized dashboard [27]. The findings from our review of mHealth apps for patients with CKD have broader implications for managing other chronic diseases. The strategies used, such as reminders, gamification, and educational content, can be adapted to improve medication adherence in conditions like diabetes, hypertension, and mental health disorders. For instance, medication reminder apps have been shown to enhance adherence in diabetes management by ensuring timely insulin administration. Similarly, gamification techniques used in CKD apps can be applied to support adherence to antihypertensive medications and lifestyle modifications in hypertension. By leveraging the insights gained from CKD interventions, health care providers can develop and implement mHealth solutions that address adherence challenges across various health conditions, ultimately enhancing patient self-management and health outcomes.

Although mobile apps were designed to enhance medication adherence, the level of engagement with these apps plays a crucial role, particularly when adherence is assessed using objective self-assessment measures [29,39]. A previous study [40] revealed that the effectiveness of nonpharmacological interventions relies on the degree of adherence to the intervention itself. This implies that patients must actively engage with the app, which can ultimately lead to improved medication adherence. Interestingly, 3 out of 7 apps include reminder features for medication intake, effectively promoting adherence to the app and creating a digital placebo effect that facilitates medication adherence in patients. This concept was previously discussed in a publication by Torous and Firth [41], suggesting that mobile apps generate a digital placebo effect on patients, where beliefs about technology support and the perception of being constantly connected to health care providers contribute to clinical improvements. Therefore, future studies could investigate this digital placebo effect as a means to ensure medication adherence through app interventions.

### Limitation

The review has several limitations that warrant consideration. First, the included studies exhibited heterogeneity in their methodological designs, app features, adherence measurement methods, patient demographics, and clinical outcomes. This diversity complicates the formulation of definitive conclusions regarding the efficacy of mobile apps for improving medication adherence. Second, the review’s exclusion of non-English publications may introduce language bias, suggesting a need for future studies to incorporate non-English sources to mitigate this bias. Third, the reviewed articles were performed in different countries. This limits the generalizability of the findings. In addition, the selected studies each had their own limitations that may have impacted the results. For instance, variations in-app features and functionalities, differences in how medication adherence was measured, and the short duration of some studies might affect the consistency and applicability of the findings. Despite the acknowledged effectiveness of internet and mobile technologies in supporting patients with chronic diseases, studies specifically investigating technology’s role in CKD self-management remain scarce. Finally, after this was completed, the assessed mobile apps may undergo additional updates and potentially launch new COVID-19 apps, which were not included in the review.

### Recommendation for Future Work

The findings of this review underscore the potential of mobile apps to improve medication adherence among patients with CKD, though the impact on clinical outcomes remains an area for further exploration. The integration of features such as reminders, educational resources, and gamification appears to enhance adherence, yet translating these improvements into tangible clinical benefits such as reduced CKD progression, lower hospitalization rates, and enhanced quality of life requires additional research. Future work should focus on longitudinal studies that assess the direct correlation between app usage and clinical outcomes. Furthermore, the development of mobile apps should consider interoperability with EHR systems to facilitate seamless data integration and enhance the coordination of care. Addressing user experience from both patient and clinician perspectives will also be crucial in refining these tools. Researchers and developers should collaborate closely with health care providers to ensure that these apps are user-friendly and effectively support clinical workflows, ultimately contributing to better patient care.

### Conclusion

This systematic review delved into the influence of mobile apps on medication adherence among individuals with CKD. Of the 9 studies included in this review, 5 reported statistically significant enhancements in adherence rates. Nonetheless, reaching definitive conclusions regarding the impact of these apps on clinical health outcomes proved challenging due to the studies’ methodological diversity, variations in-app functionalities, and outcome measurement approaches.

Future research endeavors should prioritize the establishment of standardized protocols for assessing the efficacy of mobile app-based interventions in monitoring medication adherence. This approach will foster more consistent comparisons across studies and facilitate the identification of optimal app features for enhancing adherence. In addition, there is a need to refine app functionalities, focusing on user-friendliness and patient-centered design to enhance utilization rates and maximize patient benefits.

## Appendix

Multimedia Appendix 1 PRISMA checklist.

## Abbreviations

CKD: chronic kidney disease
EHR: electronic health record
mHealth: mobile health
RCT: randomized controlled trial
SMASK: Smartphone Medication Adherence Saves Kidneys

## Data Availability

The datasets generated or analyzed during this study are available from the corresponding author on reasonable request.
